# The Precipitation Law of Cu Particles in Cu-Containing Ferritic Steel

**DOI:** 10.3390/ma19061139

**Published:** 2026-03-15

**Authors:** Ruidong Huang, Huimin Zhang, Chengyuan Zhang, Zhongwang Wu, Hao Sun, Xiaolong Zhao, Yanjun Di, Hao Zhang

**Affiliations:** 1 School of Materials Science and Engineering, Inner Mongolia University of Science and Technology, Baotou 014010, China; hrd150430@163.com (R.H.); sunhao2580@163.com (H.S.); 2 Inner Mongolia Key Laboratory of New Metal Material, Baotou 014010, China; 3 Key Laboratory of Green Extraction & Efficient Utilization of Light Rare-Earth Resources, Inner Mongolia University of Science and Technology, Ministry of Education, Baotou 014010, China; 4 Jiuquan Iron and Steel Group Gansu Hongxing Hongyu New Materials Co., Ltd., Jiayuguan 735100, China; zxl85201@163.com (X.Z.); dyj150430@163.com (Y.D.); 5 Inner Mongolia Baotou Steel Pipe Co., Ltd., Baotou 014010, China; ylh150430@163.com; 6 Inner Mongolia Fengzhou Materials Co., Ltd., Baotou 014010, China

**Keywords:** Cu-containing ferritic steel continuous temperature increase, precipitation kinetics, precipitation strengthening, degree of subcooling

## Abstract

Regarding the precipitation behavior of Cu particles in steel, conventional studies have primarily focused on isothermal precipitation, which has limitations in characterizing precipitation kinetics under variable temperature conditions. For this purpose, in the present study, the Fe-3%Si-Cu alloy was selected as a model system to systematically investigate the regulation of Cu particle precipitation behavior and associated strengthening effects in a ferrite matrix during continuous heating—a process path that better aligns with practical conditions. The results indicate that, during the continuous heating process, an increase in the heating rate from 10 °C/h to 600 °C/h leads to a significant rise in the peak temperature, from 490.2 °C to 609.7 °C, while the time required to reach the peak temperature decreases substantially, from approximately 9.1 h to 19.6 min. Through TEM microstructure analysis and characterization, it is evident that rapid heating at 500 °C/h significantly promotes the high-density nucleation of B2 and 9R-Cu metastable phases while effectively suppressing particle coarsening. This results in a finely dispersed nano-Cu precipitate phase with an average particle size of 8.21 nm and a number density of 30.35 × 10^10^ cm^−2^. Under the rapid heating condition of 500 °C/h, the precipitation strengthening contribution of Cu particles reaches 501.86 MPa, significantly higher than the 451.02 MPa observed under the slow heating condition of 50 °C/h. This study, from the perspective of the coupling effect between thermodynamics (driven by undercooling) and kinetics (governed by diffusion), elucidates the kinetic behavior of Cu particle precipitation during continuous heating. It provides a novel fundamental and strengthening theory in the field of ferrite metallurgy for copper-enriched electrical steels and related engineering steels, offering significant insights for further understanding the role of copper in ferrite-based steels.

## 1. Introduction

In recent years, the rapid development of high-speed rail, aerospace, marine, and new energy sectors has imposed increasingly stringent demands on material performance. Alloying treatment, as a key approach to enhancing material properties, has thus attracted sustained and extensive attention [1,2,3]. Copper, as a significant alloying element, plays a dual role in steel: on the one hand, it enhances process stability and formability by contributing to solid solution strengthening during high-temperature hot working [4,5,6]. On the other hand, aging treatment can induce the precipitation of nanoscale Cu-rich phases, thereby generating a significant precipitation strengthening effect, refining the grain size, and enhancing the strength, toughness, fatigue performance, and corrosion resistance of the steel [7,8,9]. Therefore, copper-containing steel has been extensively utilized in various alloy steel applications, including weathering steel, low-alloy high-strength steel, and antibacterial stainless steel [10,11,12]. Research on copper-containing steel primarily emphasizes its precipitation strengthening behavior. The precipitation kinetics of copper-rich phases significantly influence the final mechanical properties of the steel. However, the precipitation behavior of copper within ferritic matrices remains insufficiently understood and requires further investigation. Especially for Fe-Si-Cu series alloys, the single-phase ferritic structure offers an ideal model for investigating copper precipitation behavior, enabling the elimination of interference from phase transformations or complex microstructural features.

Numerous scholars have carried out extensive studies on the precipitation behavior of Cu-containing steels; however, the existing research is primarily confined to the precipitation kinetics and strengthening mechanisms of Cu-rich phases during isothermal aging. For instance, Zhang W et al. investigated the precipitation behavior of Cu particles in copper-containing ultra-low carbon steel by means of isothermal aging. It was observed that the maximum hardness (approximately 189 HV) was achieved after a short aging duration of 1 h. However, as the aging time was extended or the temperature increased, the coarsening of Cu particles resulted in a reduction in hardness. HRTEM observations were performed on the Cu precipitates following isothermal aging at 923 K (approximately 650 °C), revealing a crystal structure evolution sequence from B2 to 9R, then 3R, and finally to FCC. The contribution of precipitation strengthening to the yield strength was also quantitatively evaluated [13]. In fact, in the ferrite matrix of Fe-Cu-based alloys, the precipitation of Cu particles typically follows a definite sequence: starting from the coherently strained Cu-rich B2 (or bcc) metastable phase, it then transforms into a transitional phase with a 9R stacking order, further evolving into a 3R structure, and ultimately forming the equilibrium, non-coherent face-centered cubic (FCC) ε-Cu phase [14]. This structural evolution path is closely related to the decrease in the free energy of the system and the relaxation process of coherent strain, and has been confirmed by numerous studies using atomic probe tomography and high-resolution transmission electron microscopy. Zhang et al. systematically investigated the segregation of Cu atoms, the precipitation of nanoclusters, and the formation sequence of the ε-Cu phase during the isothermal aging of 10CrNi2Mo3Cu2V martensitic aging steel at temperatures ranging from 350 to 600 °C. They quantitatively assessed the contribution of precipitation strengthening and identified 6 nm as the critical particle size at which the Orowan mechanism becomes dominant [15]. Sharma D. K. et al. investigated the precipitation behavior of two types of quenched and tempered steels containing copper (0.6 wt.% and 1.1 wt.% Cu) under varying tempering durations by means of transmission electron microscopy and thermodynamic simulations. They found that the tempering process promoted the formation of Cu-rich precipitated phases within the equilibrium face-centered cubic (FCC) structure, and that subsequent coarsening of these precipitates led to a loss of conformality and a reduction in strength. This observation clearly established a direct correlation between the evolution of the precipitate microstructure and the tensile properties [16]. Yifan Li et al. investigated the effect of Cr content on the Cu precipitation behavior in copper-containing ferritic stainless steel during isothermal aging. They indicated that low-temperature aging promotes the formation of fine Cu precipitates, which significantly enhances the strength, whereas high-temperature aging results in coarser precipitates, thereby improving plasticity [17]. Seung-Yong L. et al. employed atom probe tomography to investigate the precipitation behavior of copper in copper-containing austenitic stainless steel during isothermal aging over the temperature range of 923 K to 1023 K. They observed that during aging at 923 K, the Cu precipitation phase initiated after 20 min, and its size progressively increased with prolonged aging time and elevated temperature. The growth behavior is consistent with the Lifshitz–Slyozov–Wagner theory [18]. However, many practical industrial heat treatment processes, such as continuous annealing and post-forging cooling, are constrained by the dimensions of the workpiece, resulting in rates of temperature change that fundamentally differ from those observed in laboratory test samples. Especially during the continuous annealing of electrical steel, the welding thermal cycle of thick-section engineering components, or the temperature-controlled cooling process of large forgings, the materials undergo a non-isothermal and continuously changing temperature history. Under such conditions, the precipitation kinetics are jointly determined by the instantaneous temperature and the heating/cooling rate. The nucleation rate, growth trajectory, and final microstructure morphology of the precipitated phases are fundamentally different from those maintained in an isothermal process at a single temperature. Therefore, establishing a precipitation kinetics model applicable to continuous temperature variations is crucial for accurately predicting and optimizing the material properties after the aforementioned industrial heat treatment. Under the heating conditions of large workpieces, variations in the heating rate significantly influence the nucleation rate, growth trajectory, and coarsening behavior of precipitated particles [19,20,21], which is fundamentally distinct from the steady-state precipitation observed under isothermal conditions. Therefore, conventional isothermal research models are inadequate for accurately describing and predicting microstructure evolution during continuous heating processes, making it imperative to establish a dedicated kinetic framework for continuous aging precipitation.

In this study, the Fe-3%Si-Cu alloy was selected as the research subject to systematically investigate the kinetic behavior of Cu particle precipitation during the continuous heating process. The core innovation of this study lies in elucidating how the key process parameter—namely, the heating rate—through the coupled effects of thermodynamics (e.g., degree of subcooling) and kinetics (e.g., atomic diffusion), synergistically governs the nucleation, growth, and spatial distribution of precipitated nano-Cu particles, thereby addressing a critical knowledge gap in the field. The research findings validate a novel mechanism governing precipitation kinetics and phase structure evolution under continuous heating conditions, establishing a dynamic analytical framework that differs fundamentally from isothermal precipitation scenarios.

## 2. Test Materials and Methods

### 2.1. Experimental Materials and Experimental Protocols

The composition range of the iron-based test steel produced in a 25 kg-scale vacuum induction furnace is presented in Table 1. The raw materials employed include high-purity iron rods (approximately 99.96%), silicon (99.999% pure), and copper (99.99% pure) (Inner Mongolia Baotou Steel Pipe Co., Ltd., Baotou, China). After melting, the resulting steel ingots are subjected to forging and hot rolling to a final thickness of 2.3 mm, followed by annealing at 980 °C for 5 min. Subsequently, the sheets are cold-rolled at room temperature to a thickness of 0.27 mm through multiple passes using a straight-pull four-high reversible cold rolling mill.

**Figure 1:**
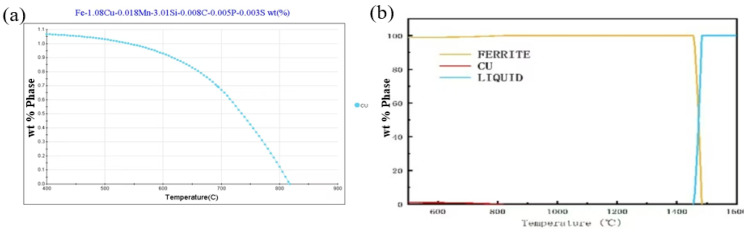
Phase diagram of Fe-3%Si-Cu alloy. (**a**) Temperature-composition phase diagram, (**b**) Phase fraction-temperature diagram.

The phase diagram of the Fe-3%Si-Cu alloy was simulated and calculated using JMatePro v13 software (Figure 1). According to Figure 1a, the solvus temperature of Cu is 807 °C. As shown in Figure 1b, with increasing temperature, only Cu particles precipitate. To achieve a uniform recrystallized microstructure and ensure complete dissolution of Cu into the matrix, the cold-rolled sample was subjected to a solution treatment at 1100 °C for 30 min. After heat treatment, the influence of recovery and recrystallization in cold-rolled samples on the hardness values can be eliminated [22].

**Figure 2:**
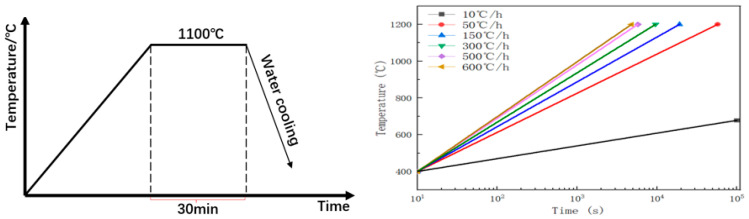
Aging experiment of Fe-3%Si-Cu alloy steel.

Combined with actual industrial production, this study aims to investigate the precipitation behavior of Cu particles in ferritic steel by designing an aging protocol for Fe-3%Si-Cu alloy steel, as illustrated in Figure 2. Below 400 °C, the kinetics of Cu particle precipitation is extremely sluggish, with virtually no precipitate formation observed. Therefore, following solution treatment, samples were heated to 400 °C at various heating rates ranging from 10 °C/h to 600 °C/h, and specimens were collected at different time intervals during the heating process, spanning from 10^1^ s to 10^5^ s.

### 2.2. Microscopic Characterization

To characterize the morphology, size, and distribution of Cu particles precipitated after continuous heating and aging, the macroscopic structure and grain morphology of the samples were examined using a Zeiss optical microscope (OM) (Axio Observer series, Carl Zeiss, Oberkochen, Germany). The hardness of samples subjected to different aging treatments was measured using an Austrian Qness fully automatic Vickers hardness tester (Q10/30/60 A/A+ series, ATM Qness GmbH, Golling an der Salzach, Austria). The distribution of second-phase particles was analyzed by means of Zeiss Sigma field-emission scanning electron microscopy (SEM) (Sigma300, Carl Zeiss, Oberkochen, Germany) and JEM-2100F high-resolution transmission electron microscopy (TEM) (JEM-2100F, JEOL, Tokyo, Japan).

The SEM sample preparation process is as follows: First, the samples were individually ground using 1000-grit sandpaper (Wuxi Gangxia Precision Abrasive Paper Factory, Wuxi, China) to ensure a surface free of visible scratches. Subsequently, mechanical polishing was performed to achieve a smooth and reflective surface. The polished samples were then subjected to electropolishing using a double A electrolyte, with parameters set at a direct current (DC) voltage of 12 V and an etching duration of 16 s in order to clearly reveal the morphology of the precipitated particles.

TEM sample preparation involves thinning the specimen to a thickness of approximately 45–50 μm, ensuring a scratch-free surface, followed by punching it into a disk with a diameter of 3 mm. Subsequently, final thinning of the specimen was performed using electrolytic double-spray polishing, employing a 10 vol% perchloric acid solution as the electrolyte. After completion of the preparation process, the sample was rinsed with anhydrous ethanol to remove surface contaminants, ultimately yielding a TEM foil suitable for high-resolution observation.

To quantitatively characterize the size distribution and surface density of the precipitated phase particles, this study conducted a systematic statistical analysis of the transmission electron microscope (TEM) images. The specific methods are as follows: Randomly select no less than 10 representative fields from each sample; use the ImageJ software (ImageJ 1.54p) for image processing and quantitative analysis. First, adjust the contrast and perform background normalization on the images. Then, set a threshold to identify the contours of Cu particles, excluding those located at the image edges or those that are obviously overlapping, to ensure the accuracy and reliability of the statistical results. To evaluate the uniformity of distribution, count the particle distribution within the crystal and near the crystal boundaries separately, and calculate their standard deviations. All statistical results are based on measurements of at least 500 particles to ensure statistical representativeness.

## 3. Test Results and Analysis

### 3.1. Aging Hardness Variation Curves at Different Heating Rates

**Figure 3:**
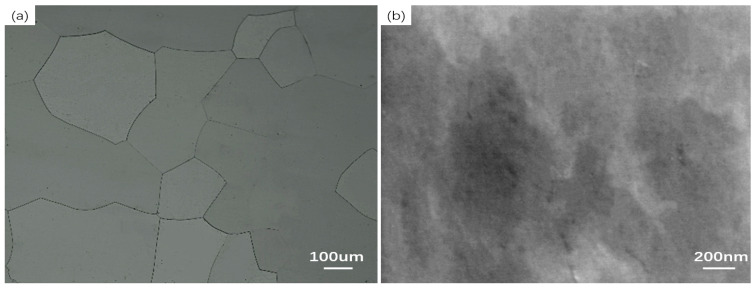
Microstructure and SEM morphology of Fe-3%Si-Cu alloy steel after solution treatment: (**a**) metallographic structure; (**b**) SEM morphology.

**Figure 4:**
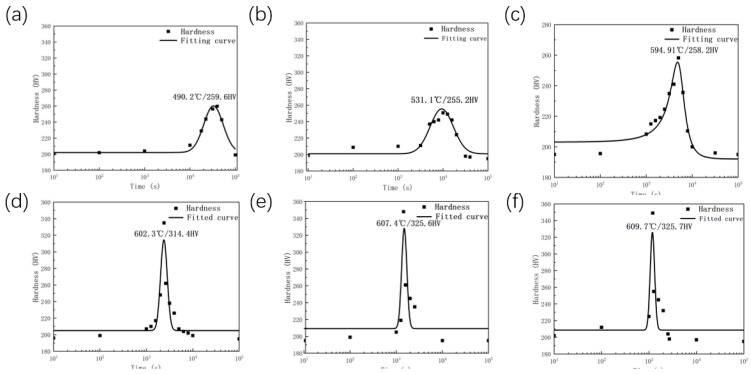
(**a**) 10 °C/h; (**b**) 50 °C/h; (**c**) 150 °C/h; (**d**) 300 °C/h; (**e**) 500 °C/h; (**f**) 600 °C/h.

Figure 3a presents the metallographic structure of the sample after solution treatment. It can be observed that the sample exhibits a single-phase ferrite microstructure, with an average grain size of 222.28 μm. Figure 3b displays the SEM morphology within the grains prior to aging. As shown, the Cu particles have been fully dissolved into the matrix after solution treatment, and no evident Cu particle precipitation is detected. According to the experimental setup illustrated in Figure 2, the aging hardness evolution curves of Fe-3%Si-Cu steel at various heating rates, presented in Figure 4, were obtained. This set of curves elucidates the influence of heating rate on the kinetics of copper precipitation. Figure 4 shows the aging hardness curves of Fe-3%Si-Cu steel at different heating rates.

Figure 4 presents the aging hardness evolution curves of Fe-3%Si-Cu steel under different heating rates (10–600 °C/h). All hardness profiles in subgraphs (a–f) exhibit a characteristic trend of initial increase, reaching a peak, followed by a gradual decrease, indicating that the material undergoes significant precipitation strengthening during the early stages of heating, followed by over-aging softening as the temperature rises [23].

To examine the effect of the heating rate, Table 2 presents the corresponding key peak parameters. When the heating rate is 10 °C/h, it takes 32,722.94 s (approximately 9.1 h) to reach the peak hardness of 259.6 HV at a temperature of 490.2 °C. The time required to reach the peak temperature is significantly reduced to 9353.43 s (approximately 2 h and 36 min) at a heating rate of 50 °C/h, with the peak temperature increasing to 531.1 °C and the peak hardness reaching 255.2 HV. When the heating rate is increased to 300 °C/h, the time to attain the peak temperature is further shortened to 1846.15 s (approximately 58 min), while the peak temperature rises to 594.5 °C and the peak hardness significantly increases to 308.7 HV. When the heating rate is further increased to 600 °C/h, the peak time is reduced to 1175.08 s (approximately 19.6 min), the peak temperature increases to 609.7 °C, and the peak hardness reaches a maximum of 325.7 HV. As the heating rate increases, the time required for the material to attain peak hardness decreases sharply, the peak temperature shifts markedly to higher values, and the peak hardness exhibits a general increasing trend.

### 3.2. Precipitation of Cu Particles When Peak Hardness Is Reached at Different Heating Rates

To investigate the microscopic mechanism of material strengthening induced by Cu particle precipitation, TEM observations were performed on samples exhibiting peak hardness under various processing conditions.

**Figure 5:**
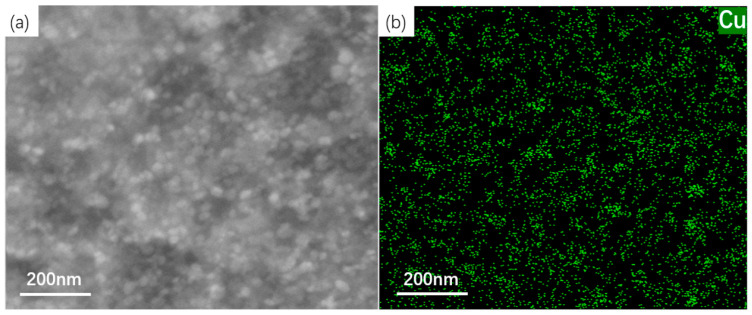
Peak hardness at aging of Fe-3%Si-Cu alloy steel: (**a**) Cu particle precipitation morphology under STEM channels; (**b**) EDS surface scan image.

Figure 5 characterizes the precipitation behavior of Cu particles in an Fe-3%Si-Cu alloy steel at peak aging hardness, as revealed by scanning transmission electron microscopy (STEM) and energy-dispersive X-ray spectroscopy (EDS) (ULTIM MAX, Oxford Instruments, High Wycombe, UK). Figure 5a clearly illustrates the distribution morphology of Cu particles within the ferrite matrix under STEM imaging, indicating that the nanoscale precipitates are highly dispersed, with no significant agglomeration observed. The EDS surface scan image in Figure 5b further confirms the chemical composition of the precipitated phase through elemental distribution analysis. Combined with contrast comparison, it is verified that these particles are Cu-rich phases, and no interference from other secondary phases is observed, indicating that Cu is the sole precipitated strengthening phase.

**Figure 6:**
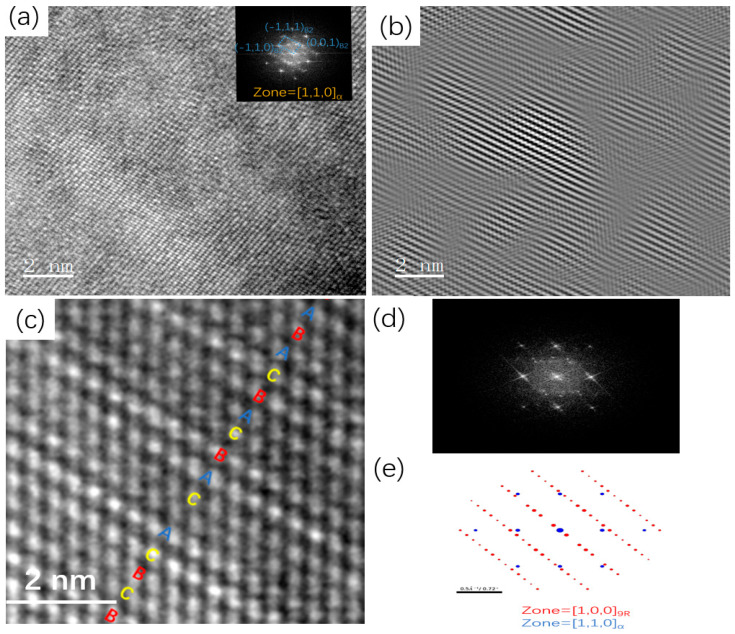
(**a**) High-resolution microscopic image of B2-Cu; (**b**) Matrix [110]α crystal band axial diffraction spots; (**c**) high-resolution microscopic image of 9R-Cu; (**d**) Matrix [110]α crystal band axial diffraction spots; (**e**) PTCLab simulation image.

Figure 6 analyzes the crystal structure characteristics of Cu particles precipitated during the continuous heating aging process, as revealed by HRTEM images. At least two distinct types of diffraction spots from the precipitates were observed in the macroscopic diffraction pattern. Figure 6a presents the HRTEM image of a precipitated Cu particle, where the matrix aligns along the <110> zone axis, and the corresponding FFT diffraction patterns of the precipitates are clearly visible. Based on the analysis of the FFT patterns and the associated IFFT reconstructions (Figure 6b), it can be concluded that the structural characteristics of this precipitate are consistent with those of Cu particles exhibiting a B2 structure [24]. Figure 6c presents the HRTEM image of another Cu particle precipitate observed along the matrix <110> zone axis. It can be clearly seen that the Cu particles arrange into a periodic structure exhibiting a 9R stacking sequence of ABCBCACAB. Its FFT spots (Figure 6d) correspond well with the diffraction spots in this orientation simulated by the PTCLab software (PTCLab-1.19) (Figure 6e), indicating that the other particle is a Cu particle with a 9R structure [25].

**Figure 7:**
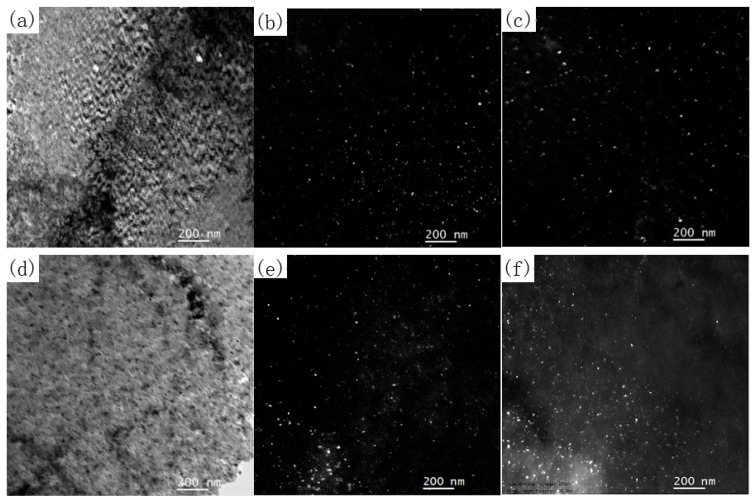
(**a**) Bright field image at peak hardness at 50 °C/h. (**b**) Dark field image of B2-Cu phase at 50 °C/h. (**c**) Dark field image of 9R-Cu phase at 50 °C/h. (**d**) Bright field image at peak hardness at 500 °C/h. (**e**) Dark field image of B2-Cu phase at 500 °C/h. (**f**) Dark field image of 9R-Cu phase at 500 °C/h.

To investigate the differences in the precipitation behavior of B2 and 9R structured Cu particles under different thermal processing conditions, this study selected samples that reached the peak hardness under representative slow (50 °C/h) and fast (500 °C/h) heating conditions and conducted micro-area bright and dark field imaging analysis and quantitative statistics. These three rate points systematically cover the continuous heating process from near equilibrium to significant non-equilibrium. Figure 7, which takes 50 °C/h and 500 °C/h as examples, shows typical microstructures. Based on the systematic image statistical analysis (with each condition involving no less than 500 particles), clear evolution patterns were revealed: as the heating rate increased from 50 °C/h to 500 °C/h, the average size of the B2-Cu phase gradually decreased from 8.64 nm to 8.43 nm, while its density increased from 10.11 × 10^10^ cm^−2^ to 11.70 × 10^10^ cm^−2^. The most significant change was in the metastable 9R-Cu phase: its density increased sharply with the increase in heating rate, from 2.222 × 10^10^ cm^−2^ at 50 °C/h, to 6.83 × 10^10^ cm^−2^ at 300 °C/h, and then significantly to 8.11 × 10^10^ cm^−2^ at 500 °C/h, an increase of over 3.6 times. Simultaneously, its average size decreased slightly from 7.33 nm to 7.055 nm. This series of quantitative data indicates that rapid heating strongly promoted the precipitation of high-density, fine metastable 9R-Cu phases.

Under a heating rate of 500 °C/h, a significant increase in the relative amount of the 9R-Cu phase is observed. This phenomenon can be attributed to the coupled effects of thermodynamics and kinetics: rapid heating induces a pronounced delay in the actual nucleation temperature, thereby resulting in an increased degree of subcooling (ΔT). According to classical nucleation theory, an increase in the heating rate leads to a broader temperature range being covered per unit time [26], which in turn shortens the diffusion time of Cu particles. This results in a significant delay of the actual nucleation temperature of precipitated Cu particles relative to the equilibrium temperature, thereby enhancing the degree of undercooling (ΔT) during the process. The increase in the degree of undercooling will reduce the critical nucleation work. This lowers the energy barrier for the formation of the metastable 9R phase, thereby facilitating high-density nucleation of the 9R-Cu phase [27]. Meanwhile, rapid heating allows the alloy to swiftly traverse the low-temperature region, thereby reducing the residence time of the precipitated phase within this zone. This suppresses the sufficient growth and coarsening of the B2-Cu phase, which is closer to the equilibrium state, thus promoting the retention of the metastable 9R-Cu phase and enabling the formation of high-density precipitates [28].

The degree of undercooling serves as the primary driving force for precipitation, and its variation directly governs the nucleation difficulty and the resulting nucleus size [29]. The nucleation process must overcome the energy barrier arising from interfacial energy [30]. The critical nucleation work, ΔG*, corresponds to the maximum energy barrier that must be overcome during the formation of such a critically sized nucleus [31]. The quantitative relationship between the two, as well as the extent of undercooling, is governed by classical nucleation theory [32]:

(1) $$d^{*}=\frac{2\sigma T_{m}}{L_{m}\Delta T}$$

(2) $${\Delta G}^{*}=\frac{16\pi \sigma^{3}T_{m}^{2}}{3{\left(L_{m}\Delta T\right)}^{2}}$$

In the equation, *σ* denotes the surface energy, *Tm* represents the melting point of Cu, and *Lm* refers to the latent heat of fusion. It can be observed from the above formula that an increasing heating rate leads to a greater undercooling, resulting in a smaller critical nucleation radius d* and lower critical nucleation energy ΔG*.

However, it is important to note that classical nucleation theory is based on idealized assumptions, including homogeneous nucleation, steady-state diffusion, and the neglect of internal stress fields. In the continuous heating process of the Fe-Si-Cu alloy studied here, particularly under rapid heating conditions, the actual precipitation kinetics may deviate significantly from predictions based on classical theory. This deviation primarily stems from the complex interplay of thermodynamic and kinetic factors: firstly, the non-isothermal nature of rapid heating and any associated transformation stresses can alter the chemical potential and diffusion barriers of solute atoms, thereby modifying nucleation sites and energy barriers. Secondly, crystal defects such as dislocations and grain boundaries can serve as potent sites for heterogeneous nucleation, substantially reducing the critical undercooling required—a phenomenon well-documented in Fe-Cu-based systems. Furthermore, continuous heating is inherently a non-steady-state process where the transient nature of solute diffusion means nucleation rates can be governed by atomic mobility kinetics rather than by thermodynamic driving force alone. Therefore, a comprehensive understanding and modeling of Cu particle precipitation under continuous heating must account for the coupled effects of thermodynamic driving force, defect-assisted nucleation, and non-steady-state diffusion kinetics [33,34,35]. The high-density precipitation of fine B2 and metastable 9R-Cu particles observed in this study under rapid heating conditions is a direct manifestation of this thermo-kinetic coupling mechanism.

**Figure 8:**
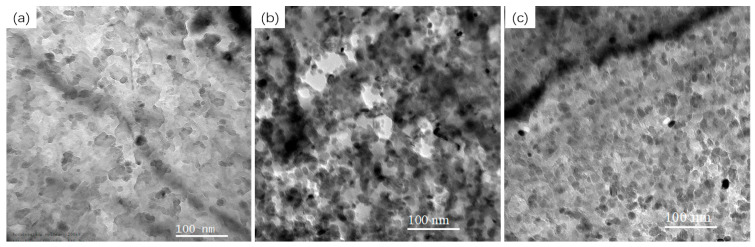
Morphology of Cu particles at the peak of aging hardness of Fe-3%Si-Cu alloy steel: (**a**) 50 °C/h; (**b**) 300 °C/h; (**c**) 500 °C/h.

To systematically reveal the influence of the heating rate on the morphology of Cu particles, this study selected three representative rate points—slow (50 °C/h), medium (300 °C/h), and fast (500 °C/h)—for detailed TEM characterization. These three conditions effectively cover the continuous heating range from near equilibrium to highly non-equilibrium, and the evolution of the precipitated phase morphology is sufficient to reveal the core laws. Figure 8 shows the typical morphology of Cu particles at the peak hardness under these three conditions: (a) 50 °C/h, (b) 300 °C/h, and (c) 500 °C/h. It can be clearly observed that as the heating rate increases, the size of the Cu particles gradually becomes finer and the distribution becomes more dispersed. For quantitative evaluation, statistical measurements of Cu particles were performed on the TEM images, and the key parameters are summarized in Table 3.

Here, *f* denotes the volume fraction of the second-phase particles, and *d* represents the average diameter of these particles in the formula. The quantity *N* refers to the number of particles in the second phase, while *A* denotes the area of the tissue under measurement [36].

(3) $$f=\frac{1.4\Pi }{6}\times \frac{Nd^{2}}{A}$$

The statistical results show that with the heating rate sharply increasing from 50 °C/h to 500 °C/h, the peak hardness temperature ranges from 538.9 to 629.4 °C, the average size of Cu particle precipitation decreases from 10.84 nm to 8.21 nm, and at the same time, its surface density significantly increases from 14.06 × 10^10^ cell/cm^2^ to 30.35 × 10^10^ cell/cm^2^.

To more clearly elucidate the advantages of the continuous heating process, this study systematically compares the experimental results with isothermal aging data reported in the literature under comparable temperature conditions. For instance, in the present work, specimens were continuously heated to approximately 600 °C at a rate of 500 °C/h (with a measured peak temperature of 607.4 °C), yielding Cu precipitates with an average size of 8.21 nm and an exceptionally high number density of 30.35 × 10^10^ cell/cm^2^. In contrast, conventional isothermal aging at 600 °C typically produces Cu precipitates in Cu-bearing ferritic stainless steels with average sizes exceeding 10 nm and number densities generally below 10 × 10^10^ cell/cm^2^. A more direct comparison further reveals that after 1 h of isothermal aging at 650 °C, Cu precipitates undergo significant coarsening, with their average size surpassing 15 nm. These comparisons collectively indicate that even when reaching similar final temperatures, the continuous heating pathway—particularly under rapid heating conditions—effectively circumvents the inevitable Ostwald ripening stage inherent to isothermal aging. The underlying mechanism lies in the large supersaturation induced by rapid heating, which strongly promotes high-density nucleation, while the limited dwell time at elevated temperatures substantially suppresses subsequent coarsening and growth. Consequently, by tuning a single processing parameter—the heating rate—it is possible to synergistically optimize both precipitate size and number density, thereby achieving superior overall precipitation characteristics compared to conventional isothermal aging.

### 3.3. Enhancement Contribution of Cu Particle Precipitation

Precipitation strengthening is one of the key strengthening mechanisms of ferritic age-hardening steels [37]. In this study, Cu acts as a single precipitate strengthening phase. An accurate assessment of its contribution requires considering the coherent state between the precipitate phase and the matrix. According to the HRTEM analysis in Figure 6, during the continuous heating process, both coherent B2-Cu phases and metastable 9R-Cu phases exist simultaneously. These two structures have different interface characteristics, and therefore their dominant strengthening mechanisms are also different.

For nano-sized precipitates that are in perfect or semi-perfect lattice matching with the matrix, their strengthening effect mainly results from the coherency strain field generated by lattice mismatch, which hinders the movement of dislocations. In this study, the B2-Cu phase and some 9R-Cu phases (especially in their early formation stage) maintain a certain degree of lattice matching with the α-Fe matrix. Therefore, we first employed the widely used coherency mismatch strengthening model to estimate their contribution [38]:

(4) $$\sigma_{Cu}=4.1MG{{\left(\varepsilon \right)}^{\frac{2}{3}}f}_{Cu}^{\frac{1}{2}}{\left(\frac{\bar{r}}{b}\right)}^{\frac{1}{2}}$$

In the formula, *M* = 3 is the Taylor factor; *G* is the shear modulus of ferritic steel, typically approximately 80 GPa; and *ε* is the mismatch parameter, taken as 0.0063; *r* represents the average radius of Cu particles; *f_Cu_* is the volume fraction of Cu; and *b* is the Bohr vector of ferritic steel, taken as 0.248 nm. The contribution of $\sigma_{Cu}$ to strength was obtained at 50 °C/h, 300 °C/h and 500 °C/h as 451.02 MPa, 496.21 MPa and 501.86 MPa, respectively. Therefore, as the heating rate increases, the grain size decreases and the strength increases, which may be attributed to the enhanced formation of the 9R-Cu phase, leading to higher Orowan strengthening.

As the heating rate increases, the proportion of the 9R-Cu phase significantly rises. As a transitional phase, the compatibility between the 9R structure and the matrix may weaken as the size increases. When the precipitated phase is non-coherent with the matrix and dislocations cannot pass through, the strengthening mechanism will transform into Orowan bypassing. To evaluate this potential contribution, we calculate Orowan strengthening:

(5) $$\sigma_{Orowan}\approx \frac{0.8MGb}{\lambda }$$

Here, λ represents the particle face spacing, which can be estimated based on the density N.

(6) $$\lambda \approx {\left(2N\bar{r}\right)}^{-\frac{1}{2}}$$

Take the condition of 500 °C/h as an example, $\bar{r}=4.11\;nm$, N = 30.35 × 10^10^ cell/cm^2^. Calculation shows that $\sigma_{Orowan}\approx 187\;MPa$, which is significantly lower than the result calculated by Formula (4) (501.86 MPa). This difference at this magnitude further supports the conclusion that the coherent slip mechanism is the main strengthening pathway at the nanocrystalline precipitate scale considered in this study.

The formation of particle precipitation involves two stages: nucleation and growth [39]. The aforementioned thermodynamic conditions govern the initiation and potential number density of nucleation events, whereas the subsequent growth rate and final precipitate size are predominantly influenced by the diffusivity of solute atoms within the matrix. Consequently, the accurate determination of the diffusion coefficient is essential for a quantitative understanding of precipitation kinetics. The diffusion coefficient quantifies the mobility of precipitating species in the matrix and exhibits an Arrhenius-type temperature dependence [40]:

(7) $$D=D_{0}exp(-\frac{Q_{m}}{RT})$$

In the equation, *D*_0_ denotes the pre-exponential factor (in units of cm^2^/s), *Q_m_* represents the activation energy for diffusion (in units of J/mol), and *R* is the ideal gas constant. For the diffusion of Cu in the ferrite matrix considered in this study, the specific expression is [41]:

(8) $$D_{Cu-\alpha Fe}=300exp(-\frac{284,000}{RT})$$

According to Formula (8), the diffusion coefficient is calculated to be 8.22 × 10^−18^ cm^2^/s at 500 °C. When the temperature rises to 550 °C, the diffusion coefficient increases to 2.80 × 10^−16^ cm^2^/s. When the temperature further rises to 600 °C, the diffusion coefficient significantly increases to 3.06 × 10^−15^ cm^2^/s. When the temperature rises from 500 °C to 600 °C, the diffusion coefficient increases by nearly three orders of magnitude, showing exponential growth.

(9) $$x\approx \sqrt{Dt}$$

In order to quantitatively evaluate the influence of atomic migration ability on the size of the precipitated phase, based on the diffusion equation (Formula (8)), we estimated the characteristic diffusion distance of Cu particles in the critical temperature range. This distance reflects the scale over which Cu atoms can migrate during the precipitation nucleation and growth stages and directly affects the final size and distribution of the precipitated phase. Under the condition of slow heating (50 °C/h), the material can remain in the temperature range of 500–550 °C for approximately 10^4^ seconds. Based on the geometric mean of the diffusion coefficient in this temperature range (1.3 × 10^−16^ cm^2^/s), the characteristic diffusion distance x is approximately 11 nm. This scale is highly consistent with the average particle size (10.84 nm) observed in the experiment, indicating that atomic migration is sufficient under these conditions, providing the necessary kinetic conditions for a significant Ostwald ripening process, which results in particle coarsening and a decrease in particle density. Under the rapid heating condition (500 °C/h), the material traverses the same temperature range at a rate of approximately 8.3 °C/min, and the residence time is shortened to 3.6 × 10^2^ s. At this point, even considering that the diffusion coefficient increases with temperature rise, the cumulative diffusion distance in this stage (500–550 °C) is only x ≈ 2.2 nm. This limited migration scale significantly inhibits aging in the low-temperature region. When the material rapidly enters a higher temperature range (such as 575–600 °C), the diffusion coefficient rises to the order of 6 × 10^−16^ cm^2^/s. Within the limited time to reach the peak temperature (about 1.5 × 10^3^ s), the characteristic diffusion distance of this stage is approximately x ≈ 30 nm. However, at this point, the high-density nucleation dominated by thermodynamic driving force (overcooling) has been completed, and the system is approaching the “frozen” state corresponding to the peak hardness. Therefore, this larger diffusion potential is mainly used to complete the distribution of atoms and short-range adjustments, rather than to trigger intense particle coarsening.

In summary, the heating rate achieves a stepwise regulation of the atomic migration scale by precisely controlling the residence time of the material in different diffusion-capable temperature zones: slow heating allows for sufficient diffusion and coarsening in the low- to medium-temperature zones, while rapid heating inhibits the maturation in the low-temperature zone and utilizes the diffusion advantage in the high-temperature zone to complete precipitation, while avoiding excessive prolonged exposure to high temperatures. This differentiated diffusion path ultimately determines the size and distribution of the precipitated phase and directly manifests as significant differences in the contribution of precipitation strengthening.

## 4. Conclusions

In this study, by systematically investigating the evolution of the structure, quantity, and size of Cu particles in Fe-3%Si-Cu ferritic steel under varying heating rates, the kinetic behavior of aging precipitation during continuous heating was elucidated. Furthermore, the influence of Cu particle precipitation on strengthening mechanisms was analyzed. The following conclusions were obtained:

(1) The heating rate predominantly governs the precipitation kinetics. As the heating rate increases from 10 °C/h to 600 °C/h, the peak temperature in the aging curve shifts from 490.2 °C to 609.7 °C, while the time to reach the peak decreases from approximately 9.1 h to 19.6 min, indicating that an elevated heating rate significantly accelerates the precipitation process and shifts the aging curve toward shorter times.
(2) The microstructural evolution of Cu particles is governed by the coupled thermodynamic and kinetic effects arising from the heating rate. The significant subcooling induced by rapid heating effectively lowers the critical nucleation energy barrier, thereby promoting high-density nucleation of fine spherical B2 and 9R-Cu metastable phases. When the heating rate is 500 °C/h, the corresponding peak temperature is 600 °C. At this time, the average size of Cu particles is 8.21 nm, and the number density is 30.35 × 10^10^ cell/cm^2^.
(3) Regulating the continuous heating rate can optimize the precipitation strengthening effect. Under rapid heating conditions (500 °C/h), the contribution of Cu particle precipitation strengthening increases significantly, from 451.02 MPa at 50 °C/h to 501.86 MPa.

## Figures and Tables

**Table 1:** Chemical composition of experimental steel (wt.%).

Element	C	Si	Cu	S	P	Mn
Content	0.008	3.01	1.08	0.003	0.005	0.018

**Table 2:** Data when hardness peaks are reached at different heating rates.

	Peak Time/s	Peak Temperature/°C	Peak Hardness
10 °C/h	32,722.94	490.2	259.6 ± 4.1
50 °C/h	9353.43	531.1	255.2 ± 6.5
150 °C/h	4996.88	594.9	258.2 ± 7.6
300 °C/h	1846.15	603.2	321.6 ± 9.1
500 °C/h	1493.28	607.4	325.6 ± 3.4
600 °C/h	1175.08	609.7	325.7 ± 6.2

**Table 3:** Parameters and volume fractions of Cu particles at peak hardness under different heating rates.

Peak Hardness at Different Heating Rates (°C/h)	Peak Temperature Tp (°C)	Average Diameter d (nm)	Surface Distribution Density N (×10^10^ cell/cm^2^)	Volume Fraction f (%)
50	538.89	10.84	14.06	8.65
300	593.98	9.47	22.30	10.47
500	629.40	8.21	30.35	10.71

## Data Availability

The original contributions presented in this study are included in the article. Further inquiries can be directed to the corresponding authors.
